# Community awareness about risk factors, presentation and prevention and obstetric fistula in Nabitovu village, Iganga district, Uganda

**DOI:** 10.1186/1471-2393-13-229

**Published:** 2013-12-10

**Authors:** Nassar Kasamba, Dan K Kaye, Scovia N Mbalinda

**Affiliations:** 1 Department of Nursing, School of Health Sciences, College of Health Sciences, Makerere University, P.O. Box 7072, Kampala, Uganda; 2 Department of Obstetrics and Gynecology, School of Medicine, College of Health Sciences, Makerere University, P.O. Box 7072, Kampala, Uganda

## Abstract

**Background:**

Obstetric fistula is a worldwide problem that is devastating for women in developing countries. The cardinal cause of obstetric fistula is prolonged obstructed labour and delay in seeking emergency obstetric care. Awareness about obstetric fistula is still low in developing countries. The objective was to assess the awareness about risk factors of obstetric fistulae in rural communities of Nabitovu village, Iganga district, Eastern Uganda.

**Methods:**

A qualitative study using focus group discussion for males and females aged 18-49 years, to explore and gain deeper understanding of their awareness of existence, causes, clinical presentation and preventive measures for obstetric fistula. Data was analyzed by thematic analysis.

**Results:**

The majority of the women and a few men were aware about obstetric fistula, though many had misconceptions regarding its causes, clinical presentation and prevention. Some wrongly attributed fistula to misuse of family planning, having sex during the menstruation period, curses by relatives, sexually transmitted infections, rape and gender-based violence. However, others attributed the fistula to delays to access medical care, induced abortions, conception at an early age, utilization of traditional birth attendants at delivery, and some complications that could occur during surgical operations for difficult deliveries.

**Conclusion:**

Most of the community members interviewed were aware of the risk factors of obstetric fistula. Some respondents, predominantly men, had misconceptions/myths about risk factors of obstetric fistula as being caused by having sex during menstrual periods, poor usage of family planning, being a curse.

## Background

Obstetric fistulas are abnormal openings that develop between the birth canal and the urinary tract (ureter, bladder and urethra) or rectum [1,2]. Most obstetric fistula follow obstructed labor, one of major causes of maternal mortality and morbidity in Sub-Saharan Africa. Such fistulas are usually associated with cephalopelvic disproportion, whereby the baby’s head presents with diameters whose dimensions are larger than the proportions of the pelvic canal through which it passes. This abnormality is associated with delays in seeking or receiving appropriate emergency obstetric care [3-8]. Obstetric fistula is an indicator of poor quality of obstetric care [9-11]. In research conducted among women with fistula in Tanzania [9-11], women who had suffered serious birth injuries such as obstetric fistulas and the health workers who looked after then found the maternity and emergency obstetric care provided inadequate [10]. Birth accounts of women with obstetric fistula suggest a health system failure in which health units fail to provide essential care to women, the health workers are disempowered, community expectations of the health system are low and rates of home deliveries are high [10,11].

Obstetric fistula is a physically and socially disabling obstetric complication that affects many women annually [11]. It has a devastating social, economic and psychological effect on the health and well-being of the affected women [11-15]. The stigma, deep sense of loss and loss of dignity and identity associated with fistula has a negative impact on quality of life [12-15]. Obstetric fistulas are one of the most distressing maternal morbidities [16,17]. In addition, they are often associated with various co-morbidities such as obstetric palsy, foot drop, renal failure, osteitis pubis, infertility, vaginal stenosis and pelvic inflammatory disease [18-21]. Furthermore, obstetric fistula mostly affects poor women, most of whom have lost their babies, during childbirth [22]. Many affected women have other complications and are subjected to social discrimination and abandonment [11,22].

Awareness of obstetric fistula is still low in many developing countries where it is prevalent [23]. In Uganda, an estimated 2.6% (about 142,000 women) of reproductive age nationally have experienced obstetric fistula [22]. In Central Eastern Uganda, 2.8% of women have obstetric fistula [22]. This estimate is probably an under-estimate, as many women with fistula do not seek treatment. Whereas the condition represents an important public health problem in Uganda, its prevalence and the level of community awareness about it are not well documented. There is limited data on awareness of obstetric fistula in communities affected by fistula, particularly its presentation, management and prevention. Misconceptions and negative beliefs might hinder seeking care for women with obstetric fistula. Low awareness might deter efforts to integrate women with fistula in their communities before and after surgery for the condition. Information on community awareness about obstetric fistula will alert health professionals and support organizations about the need for primary prevention through sensitization of rural communities about the condition. The objective of the study was to assess the level of awareness of obstetric fistula (risk factors, presentation, and prevention) among women and men of reproductive age (18–49 years) in Nabitovu Village, Iganga district. The information obtained would be used to raise awareness about obstetric fistula, to address negative attitudes and stigma directed at fistula patients. It would also be used to mobilize so that communities can support affected women’s r integration in the community before and after corrective surgery.

## Methods

### Study setting

The study was conducted in Nabitovu village, Muyira parish, Nambale Sub-county, Kigulu county, Iganga district, eastern Uganda. From the district data, the study setting (Nabitovu village) has a population of 154 households, and on average 8 people per family. It has about 1,232 people of whom about 50% are children. It was in this parish that several cases of fistula had been identified in a district survey. Most families depend on agriculture, and the major food crops are maize, potatoes and beans. The closest health units to the village are Nambale health centre III (government funded) and Nasuti health centre III which is private. These health units were used as screening centres for fistula patients, who were transferred to Iganga hospital (the district hospital) for surgery. From a previous survey [24], Iganga district has a high maternal mortality ratio of district is 397/100000, with a high fertility rate of 6.9, adolescent pregnancy rate of 37%, low women literacy rate of 48%, low family planning uptake of only 11.2%, and many cases of obstetric fistula in the sub-county. The reason for choosing the study setting was that this sub-county had the worst maternal health indicators in Iganga district [24].

### Study design and data collection

Using focus group discussions (FGDs), data was collected about awareness of obstetric fistulas. Maximum variation sampling was done to obtain a representative sample of men and women of the age group 18–49 years. Participants were identified with the assistance of the civic leaders and invited to participate. Detailed personal information of the participants was not collected. The research team, with assistance of the civic leaders, identified the venue for the meeting, identified a suitable time for the meeting, explained the purpose of the meeting. Four FGDs of 10–14 participants were conducted as follows: two for men (one for young men between 18–35 years and one for older men who are above 35 years) and two for women (one for young women between 18–35 years and one for old women between 36-49 years). Each FGD involved a moderator who guided the discussions using an interview guide and a note taker. Issues explored included risk factors, presentation and prevention of obstetric fistula. Specific issues that were probed include awareness of continuous leakage of urine as a complication of childbirth, the local meanings attached to the complication of leakage after birth, what factors predispose to or cause his complication, awareness of the management of fistula and what needs to be done to prevent obstetric fistula. The sessions lasted from 40 minutes to one hour. Each FGD member was identified with a code under which information from each was written.

### Data analysis

The data analysis was done manually by content analysis to identifying key themes, focusing on issues that were mentioned frequently and frequently received particular emphasis during the group discussions. Deductive content analysis, as described by Cavanah [24], Graneheim and Lundman [25], and Hsieh and Shannon [26]. This process involved manual identification of codes identified as meaning units (words, phrases or statements that described the phenomenon according to the issues explored regarding obstetric fistulas). The codes were aggregated into categories using a categorization matrix. After a categorization, all the data were reviewed for content and coded for connection with the identified categories. Subcategories with similar codes were finally grouped together into larger main categories or themes, according interpretation of their similarities or differences. The identified categories were compared and agreed upon by consensus, depending on their similarities. Table 1 shows the codes, categories and major categories (themes) and how they were derived from meaning units or codes.

**Table 1:** Showing categories and meaning units derived from the transcripts

**Major categories or themes**				
	**Awareness of obstetric fistula**	**Clinical presentation of obstetric fistula**	**Risk factors for obstetric fistula**	**Prevention of obstetric fistula**
**Categories**	Women deliver from home or under unskilled care get difficult delivery and genital injury	-Leaking urine	-Delivery from home	(Address birth injury)
		-Poor	-Delivery from birth attendants	-Deliver from hospital to avoid difficult delivery and therefore birth injury
			-Injury to bladder	
		-Abandoned		
**Leaking urine from childbirth injury**	Condition related to difficult childbirth	-Always wet	-Sexual violence and rape with injury to genitalia	
				-Care taken by doctors during surgical operation to ensure no bladder or genital injury
		-Must use pads		
	-Persistent smell	-Can mot move out without padding herself		
	-Woman is always wet and uses pads			
			-Operative delivery	
			-Operation by unskilled health workers	
	-Woman covers herself with sacs			
				-Deliver with a skilled attendant to avoid prolonged labor and birth injury
	-Woman leaves a patch of wetness where she has been seated			
			-Manipulation –	
			-Unskilled birth attendants	
				-Examine genital tract after delivery or operation to identify tears
			-Rude health care providers	
**Misfortune**	-Mother loses baby	-Adverse childbirth complications	-Bewitched	(Address misfortune)
			-Poverty	
			-Married young	
			-Not educated	Prevent early marriage
			-Gods unhappy	
	-Mother is isolated			
				-Address poverty
				-Educate girls
		-Difficult labor		
		-Baby dies		
	-Mother always smells urine	-Mother abandoned		
		-Mother gets permanent injury	-Rape and physical violence	
**Stigma**	Mother isolated	-Mother isolated	-Had induced abortion	Address stigma of fistula
	-Mother suffers ridicule	-Mother suffers ridicule		
			-Had sex during menses	-Address misconceptions
	-Abandoned by relatives			
	-Loses marriage	-Abandoned by relatives or family		
		-Loses marriage	-Venereal disease	-Health education campaigns targeting men

### Ethical considerations

This research was approved by the Department of Nursing and the ethics committee of the College of Health Sciences, School of Medicine, Makerere University. Permission to conduct the study was also obtained from the local council one (LC1) chairman of Nabitovu village. Confidentiality was assured and no names were written down during the discussions. Any community members who needed more information on obstetric fistula were refereed to the fistula treatment centre located at the health centre.

## Results

### Community’s awareness about presentation (symptoms and signs) of obstetric fistula

While most male participants reported that they had never heard of the condition of obstetric fistula. On probing, some participants reported that they were aware of a condition characterized by leakage of urine, persistent smell of urine following difficult delivery, or women who constantly used clothes pads to prevent leakage of urine. Locally, the condition was referred to as *“Okudhabada” or “Kayisameinhe”*. Literally, this means ‘constant leakage of urine by an unfortunate woman after giving birth’. While participants reported uncontrolled leakage of urine, there was no comment about any other leakage, such as faeces. Either the participants were not aware of this related condition or could not relate it to leakage of urine. Victims of obstetric fistula are ever uncomfortable as almost all the time, their knickers and skirts are ever wet because of the continuous leakage of urine, as exemplified by one participant:

“These women usually suffer as every time they get up, the position/seats which they had occupied before gets wet, plus their skirts, so they end up changing and washing their clothes many times in a day”.

Another participant from the older men’s focus group reported that the problem (of obstetric fistula) occurred to his own wife:

“Because of over leaking of urine, my wife used to walk with a sisal sack so that she could cover the wet skirts”.

Victims of obstetric fistula were reported to have a persistent bad odor. Due to this odor, fistula patients are always isolated by the community and close family members. They were often abandoned, and huts were constructed for them far away from the family home. If it was a married woman who happened to have the condition, the husband abandoned and got another wife:

“*My sister was (abandoned and) left in misery by the husband without any support for herself and the children, and her husband ended up marrying another wife because he could not tolerate putting up in the same bed (with my sister) because of the stench” (Participant from the older women’s focus group).*

“Some of the victims also give up on their small businesses, for example, a friend of mine used to attend to a food joint but because of the odor, which could come out of her, her customers started reducing in number up to when a time reached when no one could come around to buy her food,… so she decided to close up the business and was left with no source of income, apart from begging”. (Participant from the young women’s focus group).

Women with obstetric fistula were always depressed or in low moods due to the isolation and stigma. They had low self-esteem and had nobody to go to for help, since most of the community members isolate them.

“*Obstetric fistula victims feel lonely because of the odor and sometime they attempt to committee suicide because they reach an extent when their minds tell them that they do not have any reason to live and they are worthless” (participant from the older women’s focus group).*

### Perceived risk factors for obstetric fistula

Participants were asked a direct question: *What causes obstetric fistula-the condition in which women continuously leak urine after childbirth?* Many could not identify a specific cause, but instead mentioned perceived predisposing or risk factors. Delay to access medical care during labor, particularly for women who got complications while trying to deliver from the community, was identified by all the groups as a risk factor:

“Obstetric fistula (Okudhabada) is mainly caused by delay to be taken to a health centre if the women gets problems during delivery, or attempts to deliver at home or delivers alone by herself” (young woman FGD participant).

Delay occurred during attempts at home delivery. While at home, any one can do whatever she thought in attempts to accomplish the delivery. Such manipulations could also lead to fistula:

“(In situations where the delivery has delayed), overfixing of the hand in the birth canal by very many individuals may also contribute to”kayisameinhe” (participant from young women’s focus group).

Most of the women participants attributed obstetric fistula to young age of women. They argued that women married at a young age get pregnancies which are followed by complications such as genital injuries (during childbirth):

“Okudhabada” can be linked to young age. This is because the reproductive organs of these young women have not yet grown to capacity to allow normal delivery (process) of the baby. So as a result, the (urinary) bladder might be injured during the process of struggling to push out the baby”. (Participant from the young women’s focus group).

“Young girls who are forced by their parents to get married are usually victims of Okudhabada. Their bodies have not yet grown to capacity for ‘smooth’ delivery of their babies, …so as the baby tries to negotiate its way out, the bladder might be damaged”. (Participant from the older women’s focus group).

Induced abortions were perceived to be a major contributing factor to obstetric fistula. Induced abortions were known to exist in their community, and were performed at both the community levels and at the hospitals. They were thought to contribute to *“kayisameinhe*” through damage which might be inflicted on the bladder. Some participants thought effects of induced abortions manifest as complications during subsequent delivery as “*okudhabada”*:

“Abortions which are mainly brought about by unwanted teenage pregnancies usually leave young girls in a desperate situation of constantly leaking urine and having a bad odor after delivery”. (Participant from focus groups of young women).

Having sexual intercourse during menstrual periods was also perceived by men and women as a factor that can lead to obstetric fistula, through possible damage to the genital tissues.

“When someone has sex during their periods, they are most likely to get the problem of Okudhabada”. (Participant from focus groups of older men).

Having a caesarean section after a difficult labor was identified as a predisposing factor for obstetric fistula during the process of delivery, however the actual mechanism by which the damage leads to leakage of urine was unclear:

“*For me, am speaking from a real life situation point of view. After my sister having a difficult labor in a health unit, she was rushed to hospital where she was operated, the baby was out;… dead, and on returning home, she started leaking urine”. (Participant from young men’s focus group).*

Whereas most of the participants pointed out that women who experience difficulties in labor and are taken to theatre for operations end up leaking urine, they argued that sometimes (the mothers) come out when their bladders have been accidentally injured by the surgeons. Surgeons sometimes never notice it in time the injuries caused, so the women are discharged without the puncture on the bladder being repaired:

“Sometimes a doctor can easily make a cut on the bladder as he tries to get the baby out of the mother’s womb. This he leaves the woman with devastating effect of uncontrolled leakage of urine”. (Participant from young women’s focus group).

Some of the participants expressed a concern, that traditional birth attendants also cause the problem of obstetric fistula as they have limited skills of child birth and management of the mother after giving birth.

“*Giving birth from villages, as some of the traditional birth attendants can ask a women in labour to “push in a wrong time” so this strains the bladder which might lead to its rupture thus a woman develops a problem of kayisameinhe after giving birth”. (Participant from young men’s focus groups).*

“It can also reach a time when the traditional birth attendant frequently fixes her a hand into the birth canal of the women in labor this action can put a woman at a risk of her bladder and other organs being injure” (participant from group young women’s focus group).

However some women participants, particularly from the older women’s focus group, had a different view regarding traditional birth attendants as a cause of obstetric fistula, identifying that they were very caring to the pregnant mothers in labor.

“But sometimes we should not put too much blame on women who go to traditional birth attendants while in labor. These have experience, give them (women in labor) a warm welcome to the extent of preparing for them tea and simple food as compared to the midwives in health units who sometimes never care”. (participant from older women’s focus group).

Trained midwives at the health units were also indirectly blamed for the cause of obstetric fistula. Some women reported that when they deliver from health centres, sometimes they (midwives) ask for money before conducting a delivery and usually it is not less than 10,000/= (about 4 US dollars) and yet most traditional birth attendants ask for less or even nothing at all. Thus midwives contribute to the delay in delivery.

*“The unkind words (midwives) can ever speak out to you that you ‘do not make noise for me, it is not me who made you pregnant’ , …..so at least the midwives should change the way they handle these women in labor”. (participant from older women’s focus group)*.

*“----sometimes we don’t have money to go to health centres so we use traditional birth attendants who are our friends and above all they are near our homes….” (participant from group older women’s focus group)*.

Some participants attributed obstetric fistula to sexually transmitted diseases like syphilis. These diseases were believed to cause damage to the internal reproductive organs up to the bladder thus resulting in uncontrolled leakage of urine after delivery:

“Kabotongo (syphilis) can make a woman to get obstetric fistula because syphilis can ‘eat up’ the internal organs up to the bladder”. (participant from older men’s focus group).

Some of the participants believed obstetric fistula was not related to a biomedical cause but may be due to a curse, and that any individual who had an obstetric fistula could have been cursed by paternal aunt or gods, and therefore was considered a misfit in society:

“*Women who sometimes have wrangles/misunderstandings with their paternal aunties can be cursed by these aunties and end up with constant leakage of urine after giving birth”. (participant from older women’s focus group).*

“This is usually a sign that the gods are unhappy about certain things such as the relationship;….for example if certain cultural practices are not followed like buying a goat and a cock for lubaale (‘traditional gods’) of the clan when a woman is going to marry can cause such conditions (like fistula) after her giving birth.” (Participant from older women’s focus group).

Gender-based violence was also believed to be a predisposing factor to obstetric fistula. While some participants pointed out that domestic violence can cause obstetric fistula, the exact mechanism by which fistula occurs was not reported. However, some respondents believed that domestic violence, especially which involves physical fighting when a woman is pregnant, can severely injure the woman and the end result might be an obstetric fistula, through an exact mechanism that was not known or was not clear:

*“Fighting between man and a pregnant woman can put a woman’s life in danger, by causing some injuries, and (women) might end up with Kayisamainhe.” (participant from older men’s focus group)*.

Many participants believed that obstetric fistulas were associated with and followed sexual trauma after rape. They argued that women who are forced to have sex end up with injury that is inflicted on their urinary system, and that they end up constantly leaking urine after childbirth.

“When women are forced into sex through rape, usually men in a group use a lot of energy during the group sex or ‘Kajegere’ , thus the victims end up with injured bladder which might lead to obstetric fistula”. (participant from group young men’s focus group).

“If the girl happens to survive the direct injury after being raped, she might not survive getting pregnant and she might get difficulties during delivery of the baby. Although some girls go in for abortions when they get to know that they are pregnant after being raped, they might still get the condition of obstetric fistula” (participant from young women’s focus group).

Some participants also blamed use or misuse of family planning methods as a contributing factor:

“When women who use family planning methods, especially tablets, miss to take some of their tablets in time, they risk getting ‘okudhabada’ (obstetric fistula)”. (participant from young men’s focus group).

### Treatment of obstetric fistula

The most effective way of treating obstetric fistula was perceived to be through surgical operations. Early detection of the condition and hospitalization usually yielded positive results as most of the fistula victims usually recovered completely. These women (after surgery) got integrated back into the community with the help of health workers, their families and other stake holders and were able to resume with their former day to day activities:

“After my sister being operated by a team of visiting surgeons, under Uganda village project, she first spent some time at Kamuli mission hospital for monitoring by the health workers then when she came back home, she had stopped leaking urine and the odor had also reduced so much”. (participant from focus young women’s focus group).

Certain herbs provided by the traditional healers usually reduced the leakage of urine although they could not “seal the puncture” on the bladder:

“Mvule tree roots are usually crushed into powder form and given to a woman to smear in a piece of cloth which she fixes around her private parts”. (Participant from young women’s focus group).

However, some participants had different views, and doubted if herbal medicine had any value:

“I swear no herb can completely seal off a puncture that causes ‘kayisameinhe’ , unless a patient with the condition goes to hospital and it is surgically sealed off with threads”. (participant from the older women’s focus group).

### Preventive measures for obstetric fistula

Careful operations on pregnant mothers by surgeons should be emphasized. After removal of the baby, thorough “examination” of internal organs should be done in order to rule out the “cuts” that might have been made on the bladder and corresponding tissues which might lead to an obstetric fistula:

“If any cut/puncture is found out by the surgeon, it should be attended to in time in order to save the woman from the shameful condition of okudhabada.” (Participant from focus older men’s focus group).

Some participants argued that one of the reasons as to why early marriage is common is poverty, so some families look up to their daughters as a source of income so they are married off at an early age. Discouraging early marriages would greatly contribute to the prevention of obstetric fistulas:

“If we are to do something about this problem then we should keep our young girls in school so that the chances of getting into contact with men to deceive them around are reduced. We should also request teachers to keep on counseling our daughters and advice them accordingly to enable them stay focused on their studies”. (participant from older women’s focus group).

Some participants believed that when pregnant women in labor sought early medical attention, chances of getting obstetric fistula would be greatly reduced.

“Although sometimes some women have difficulties getting to the health centers for delivery because of (especially) financial reasons, I personally, the day I get pregnant (I will) start saving at least a coin with my husband every day such that when labor time reaches, money for transport to the health unit and other small necessities are not a problem to acquire”. (participant from older women’s focus group).

## Discussion

The study shows that many participants had low awareness about the condition, it causes or its prevention, with a lot of misconceptions. Prolonged obstructed labor without appropriate obstetric emergency care is the main causes of obstetric fistula. Therefore, many community members had correct information regarding some of the risk factors of obstetric fistula, but were unaware of the mechanism by which fistula occurs or had misconceptions on the cause of obstetric fistulas. The participants correctly attributed obstetric fistula to delay in seeking early medical attention by a woman in labor, particularly in situations where there were obstetric complications. While the lack of awareness of obstetric fistula may be true, the purported ignorance could be due to the fact that many communities do not want to reveal existence of the condition in their community. Due to the stigma associated with the condition, many people in rural communities might pretend ignorance of the condition. For instance, previous research has showed that there is a tendency of some people not wanting to reveal the existence of obstetric fistula in their community [27].

The community was also aware of come conditions that are risk factors for obstetric fistula, though might not necessarily be predisposing factors or causes of obstetric fistula. Early marriages, induced abortions, use of traditional birth attendants, rape, domestic violence and caesarean sections (operations) are predisposing factors for obstetric fistula [28]. Pervasive poverty is an important cause of fistula [29-31]. Likewise, women who suffer from obstetric fistula tend to be impoverished, malnourished, lack basic education and live in remote or rural areas [29-31]. The fact that the community attributed the cause of fistula to negligent or inexperienced doctors, or to negative attitudes of health workers may negatively affect health-seeking behavior for obstetric care by expectant mothers in labor, including those with difficult deliveries. Similar findings have been reported in a study in Tanzania [10,11], whereby (real or imaginary) bad birth care experiences might undermine the reputation of the health care system, lower community expectations of facility birth, and consequently reduce health facility births. Such mothers might assume that whoever goes to theatre for emergency operations (like caesarean section) has high chances of ending up with the condition. This would be unfortunate, since it is failure or delay to address the childbirth complications that is responsible rather than the operation to relieve the complications.

Despite having correct knowledge concerning risk factors for obstetric fistula, many community members had misconceptions about obstetric fistula and attributed the condition to curses, cultural spirits (considered the condition as a curse from gods), sexually transmitted disease, having sex during menstrual periods and improper use of family planning methods. Such misconceptions on aetiology of obstetric fistula are common in many rural communities. In a study by Kazaura et al. [32], missing family planning tablets for some days was associated with obstetric fistula. Likewise, cultural spirits and having sex during menstrual periods were reported as contributing to obstetric fistula [32]. A study on perceived causes of obstetric fistula in Nigeria [33] reported spiritual causes as one of the perceived risk factors for obstetric fistula. Certain cultural beliefs related to childbirth, social stigma attached to getting a cesarean section delivery, preference for delivering with traditional birth attendants, and perception of unpleasant and abusive experiences during delivery (regarded to be prevalent in many medical centers) usually force women to go to traditional birth attendants [32,33]. Though such women are referred to hospital if health workers identify or anticipate complications, the preference for avoiding health centers or hospitals may be so strong that the mother will merely seek out another traditional birth attendant. In that way, the risk of obstetric fistula is aggravated.

Many non-obstetric factors are perceived as predisposing factors for obstetric fistula in different communities. These include young and short in height [6], marginalization [34], or low level of education, female genital mutilation, spiritual causes, sexual abuse, young age at marriage and being unmarried [33]. Others are low education, poverty, rural residence and sexual violence [35]. While the immediate causes of obstetric fistula in developing countries are obstructed labor and lack of prompt access to emergency obstetric care, pervasive poverty is often the root cause [30,31]. Many patients with fistula come from remote areas and are of low economic status [30,31]. The risk factors for obstetric fistula could be analyzed using the framework of the 3 delays, adapted from the framework on determinants of maternal mortality [36]. In such patients, lack of awareness about danger signs of obstructed labor causes the first delay to receive prompt care. This first delay is compounded by lack of autonomy in decision-making and misconceptions about healthcare delivery, which negatively affect health-seeking behavior in case obstetric complications. Absence of emergency transport or lack of money to meet transport costs constitutes the second delay to receiving prompt care in case of emergency referrals. Delay or failure to receive prompt care constitutes the third delay. The incidence of obstetric fistulae is therefore closely related to culture, socio-economic status and level of education of individuals in a given setting [37-39].

Awareness of obstetric fistula in developing countries remains a great challenge, and inadequate resources contribute greatly to this problem. Indeed, even patients often have limited knowledge about their condition. For instance, in a review of causes of obstetric fistula from patients’ perspective [38], 70% of the women attributed obstetric fistula to prolonged labour, while 33% attributed the condition to God or operation to correct obstructed labor. This study revealed that pre-operative and post-operative counseling services were inadequate in correcting the levels of low community awareness of the risk factors and symptoms of obstetric fistula. In another community study from Nigeria [40] found low level of awareness of obstetric fistula, as of 130 patients with fistula interviewed, only 70% could identify the cause as prolonged labor. Similarly, a study from Cameroon showed that 41.7% of women with fistula had no knowledge of the cause [41]. There was modest awareness of the signs and symptoms of obstetric fistula as participants pointed out uncontrolled leakage of urine and unpleasant odor as the remarkable signs. However, participants were not aware of leakage of stool associated with some fistulas. Likewise, participants were not aware of other common signs and symptoms or associated complications of obstetric fistula. These include recurrent vaginal infections, neurological damage like foot drop and contracture of the lower limb, vaginal scarring and sexual problems, amenorrhea and infertility [10,28].

### Perceived management of obstetric fistula

There was a misconception about fistula treatment as some participants believed that certain herbs could be used to cure obstetric fistula. This misconception could be explained by the low awareness of the problem of obstetric fistula in the community. The findings are encouraging that most participants recommended surgical repair of the fistula as the treatment for fistula. The low awareness about fistula treatment could be explained by the small number of hospitals that have skilled personnel and equipment for surgical repair obstetric fistula.

### Perceived prevention of obstetric fistula

The participants had good knowledge on the preventive measures of the condition and these included careful operations, discourage early marriages, health education campaigns, early seeking of medical attention during labour, easy access to health units and safety of young girls. The participants’ inadequate knowledge about preventable measures highlight the need for improved monitoring of labor, improved access to emergency obstetric services (particularly cesarean delivery), competent medical care for women both during and after obstructed labor, and the development of specialist fistula centers to treat injured women where fistula prevalence is high.

## Conclusion

Many women and a few men in Nabitovu village are aware of the presentation and risk factors of obstetric fistula despite certain misconceptions held some individuals about the condition, particularly what causes it. The participants’ view emphasizes the fact that long-term strategies to eradicate obstetric fistula must include more awareness of the condition, its presentation and risk factors. Secondly, there is need for improved access to family planning services, increased education for girls and women, community economic development, and enhanced gender equity and as well as mobilization of all stakeholders to ensure that adequate resources are devoted to this problem.

## Competing interests

The authors declare that they have no competing interests.

## Authors’ contributions

DKK, NK and SNM conceptualized the study. NK conducted the data collection. DKK and NK conducted the data analysis. DKK wrote the text of the paper. All the co-authors gave advice on presentation of the results and editing of the text, and approved the final manuscript.

## Pre-publication history

The pre-publication history for this paper can be accessed here:

http://www.biomedcentral.com/1471-2393/13/229/prepub
